# HIF in Nephrotoxicity during Cisplatin Chemotherapy: Regulation, Function and Therapeutic Potential

**DOI:** 10.3390/cancers13020180

**Published:** 2021-01-07

**Authors:** Siyao Li, Lu Wen, Xiaoru Hu, Qingqing Wei, Zheng Dong

**Affiliations:** 1 Department of Nephrology, The Second Xiangya Hospital of Central South University, Hunan Key Laboratory of Kidney Disease and Blood Purification, Changsha 410011, China; lisiyao0716@csu.edu.cn (S.L.); 188201019@csu.edu.cn (L.W.); 188201029@csu.edu.cn (X.H.); 2 Department of Cellular Biology and Anatomy, Medical College of Georgia at Augusta University, Augusta, GA 30912, USA; qwei@augusta.edu; 3 Charlie Norwood VA Medical Center, Augusta, GA 30912, USA

**Keywords:** cisplatin, HIF, hypoxia, acute kidney injury, chronic kidney disease, nephrotoxicity

## Abstract

**Simple Summary:**

Cisplatin is a widely used chemotherapy drug, but its use and efficacy are limited by its nephrotoxicity. HIF has protective effects against kidney injury during cisplatin chemotherapy, but it may attenuate the anti-cancer effect of cisplatin. In this review, we describe the role and regulation of HIF in cisplatin-induced nephrotoxicity and highlight the therapeutic potential of targeting HIF in chemotherapy.

**Abstract:**

Cisplatin is a highly effective, broad-spectrum chemotherapeutic drug, yet its clinical use and efficacy are limited by its side effects. Particularly, cancer patients receiving cisplatin chemotherapy have high incidence of kidney problems. Hypoxia-inducible factor (HIF) is the “master” transcription factor that is induced under hypoxia to trans-activate various genes for adaptation to the low oxygen condition. Numerous studies have reported that HIF activation protects against AKI and promotes kidney recovery in experimental models of cisplatin-induced acute kidney injury (AKI). In contrast, little is known about the effects of HIF on chronic kidney problems following cisplatin chemotherapy. Prolyl hydroxylase (PHD) inhibitors are potent HIF inducers that recently entered clinical use. By inducing HIF, PHD inhibitors may protect kidneys during cisplatin chemotherapy. However, HIF activation by PHD inhibitors may reduce the anti-cancer effect of cisplatin in tumors. Future studies should test PHD inhibitors in tumor-bearing animal models to verify their effects in kidneys and tumors.

## 1. Introduction

Cisplatin is an effective and broad-spectrum chemotherapeutic agent for various kind of tumors, such as testicular, ovarian, head and neck, bladder, lung, breast, and cervical cancer [1,2]. Though being used worldwide, the therapeutic efficacy of cisplatin is limited, to some extent, by its side effects in normal tissues including ototoxicity, neurotoxicity, and nephrotoxicity [3]. Among them, cisplatin-induced kidney injury or nephrotoxicity is life-threatening and has attracted great attention with numerous studies focusing on its underlying mechanisms and potential therapeutic strategies [4,5]. Cisplatin can induce both acute kidney injury (AKI) [5,6] and the chronic kidney diseases (CKD) following AKI [7,8,9].

Hypoxia is a common factor involved in the development of renal pathology in both AKI and CKD [10]. Hypoxia-inducible factor(HIF), firstly discovered in the studies of erythropoietin(EPO), is generally considered as the core regulator for maintaining oxygen homeostasis due to its crucial role in cellular sensing and adaption to hypoxia [11,12,13]. As a transcription factor, HIF binds to DNA in a sequence-specific manner to promote or repress the transcription of multiple genes. HIF is widely involved in various biological processes, such as oxygen sensing, angiogenesis, vasodilation, erythropoiesis, metabolism, inflammation, and cell-cycle regulation [14,15]. Several studies have reported that HIF may participate in cisplatin-induced nephrotoxicity. However, most of these studies focus on cisplatin-induced AKI and find that HIF activation may protect against tubular cell injury and promote kidney recovery [16,17,18,19]. The lack of information of HIF in AKI to CKD progression and chronic kidney problems following cisplatin exposure make it an urgent need to study the regulation of HIF in these conditions.

Hypoxia is a known condition in solid tumors due to abnormal cancer cellular proliferation, expansion of tumor size and disruption of angiogenesis. As such, HIF is activated in tumors for cellular adaptation, and studies have found that inhibition of HIF may be an anti-cancer strategy under some circumstances [20]. In cisplatin chemotherapy, the activation of HIF for kidney protection may therefore antagonize the anti-tumor effects. Thus, when considering HIF activation as a kidney-protective strategy in cisplatin chemotherapy, the effect on cisplatin’s anti-tumor efficiency has to be taken into consideration.

In this review, we briefly introduce the major characteristics of HIF and cisplatin-induced nephrotoxicity, summarize the studies investigating how HIF participates in the renal pathological process after cisplatin exposure, and propose future research directions pertaining to HIF in cisplatin-induced AKI and CKD. We especially discuss the possible therapeutic strategies by activating or targeting HIF for cisplatin-induced nephrotoxicity as well as their influences on the anti-cancer effect of cisplatin.

## 2. Characteristics of HIF

### 2.1. Structure of HIF

HIF is a protein heterodimer complex of two subunits, HIF-α and HIF-β [12,21]. While HIF-α expression is oxygen sensitive and induced by hypoxia, HIF-β is constitutively expressed. HIF-α has three isoforms, ie. HIF-1α, HIF-2α and HIF-3α. HIF-1α is widely expressed in various cell types, while HIF-2α is mainly found in some specific cell types, such as vascular endothelial cells, renal interstitial cells, type II pneumocytes, liver parenchymal cells, and cells of the myeloid lineage [22,23,24]. HIF-3α is mainly found in the thymus, cerebellar Purkinje cells, and corneal epithelium of the eye [25,26,27]. HIF-β is stably expressed in an oxygen-independent manner in most cell types [13,28]. The HIF-α subunit binds to HIF-β to form the corresponding members of the HIF family. Although both known as essential regulators of hypoxia-induced transcriptional activities, HIF-1 and HIF-2 may have some differences in their roles. While HIF-1 is mostly reported to maintain the initial adaptation to hypoxia, HIF-2 mainly works under the long-term hypoxic conditions [29]. HIF-3α has various splice forms, which may either negatively or positively regulate the transcription activities of HIF-1 and HIF-2, and the function of these splices remains largely unclear [26,27,30].

Both HIF-α and HIF-β share the basic helix-loop-helix-PER-ARNT-SIM (bHLH/PAS) domains at their N-termini, by which the two subunits heterodimerize with each other and bind to specific DNA sequences [31]. All isoforms of HIF-α and HIF-β contain the transactivation domain (TAD) at their C-termini for transcription regulation, whereas HIF-1α and HIF-2α also have N-terminal TAD (N-TAD) [32]. Notably, HIF-1α and HIF-2α contain an oxygen-dependent degradation domain (ODDD) that mediates oxygen-induced inactivation and degradation.

### 2.2. Regulation of HIF

As HIF-β is stably expressed, the regulation of HIF expression and function mainly depends upon HIF-α. HIF-α can be modulated at multiple levels including transcriptional, translational, and post-translational regulations.

At the transcriptional level, HIF-α mRNA expression may be induced by hypoxia or ischemia [13]. Nuclear factor kappa B (NF-κB) pathway is reported to regulate HIF-α mRNA transcription directly [33]. Yamaguchi et al. [34] further reported that NF-κB pathway regulates CCAAT/enhancer-binding protein δ(CEBPD), which may directly promote HIF-1α transcription by binding to its promoter in both acute and chronic hypoxic kidneys.

At the translational level, although the overall protein synthesis is suppressed during cellular hypoxia, the synthesis of HIF-1α and HIF-2α is not reduced [13]. Stimuli such as oncogene activation, tumor suppressor inactivation, growth factors, and varieties of cytokines may also promote HIF-α expression [35]. Growth factor heregulin has been reported to induce HIF-α translation to maintain the balance between oxygen consumption and delivery [36]. Recent studies have shown that microRNAs also contribute to the regulation of HIF-α expression [37,38,39].

**Figure 1:**
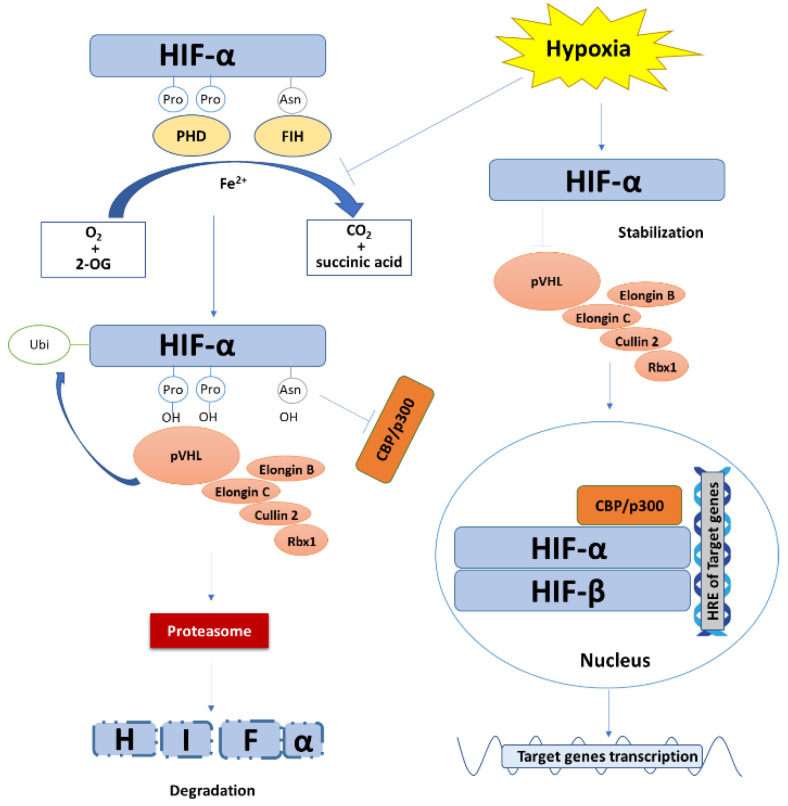
The degradation, stabilization, and transcriptional function of HIF. Under normoxic condition, with Fe^2+^ as a cofactor and oxygen and 2-oxoglutaric acid (2-OG) as substrates, PHD and FIH hydroxylate HIF-α at the site of two proline residues and an asparagine residue in ODDD, respectively, accompanied by the generation of carbon dioxide and succinic acid as by-products. The prolyl hydroxylated HIF-α is preferentially recognized and ubiquitinated by VCB-Cul2 E3 ligase complex and is then targeted to proteasome for degradation. Meanwhile, hydroxylation of the asparagine residue of HIF-α prevents its recruitment and binding to coactivator p300 and CBP. Under hypoxic stress, the hydroxylation reaction of PHD and FIH is inhibited. The VCB-Cul2 E3 ligase complexes are not able to recognize and ubiquitinate HIF-α, leading to the stabilization and accumulation of HIF-α. HIF-α then translocates into the nucleus, heterodimerizes with HIF-β, and recruits coactivator p300 and CBP to specifically bind to the hypoxia response elements (HRE) of target genes, resulting in gene transcription.

Nonetheless, the most critical mechanism regulating HIF-α expression is the post-translational degradation (Figure 1). In the presence of oxygen, HIF-1α or HIF-2α are hydroxylated at the sites of two proline residues (P402/P564 and P405/P531 for HIF-1α and HIF-2α in human, respectively) in ODDD domain by prolyl hydroxylase (PHD). The hydroxylated HIF-α is preferentially recognized by the Von Hippel-Lindau tumor suppressor protein (pVHL) [40]. pVHL binds to Elongin C and then forms the VCB-Cul2 E3 ligase complex that is composed of pVHL and elongin C, elongin B, cullin-2, and Ring-Box 1 (Rbx1). Thereby, HIF-α is ubiquitinated by the VCB-Cul2 E3 ligase complex and is targeted to proteasome for degradation [41,42]. Besides, the existence of oxygen also enables the enzymatic activity of factor inhibiting HIF-1 (FIH). FIH hydroxylates an asparagine residue (N803 in huamn HIF-1α) in the C-terminal of HIF-α and prevents the recruitment and binding of HIF-α coactivator p300 and CREB-binding protein (CBP) [43]. In hypoxia, the lack of oxygen prevents the hydroxylation response of PHD and, as a result, HIF-α is not ubiquitinated for degradation, leading to the accumulation of HIF-α protein. In this regard, PHD inhibitors have received intensive interests for their potent ability of inducing HIF [44,45]. Several oxygen-independent mechanisms for HIF stabilization are also involved in the regulation of HIF-α. Receptor for activated C kinase 1 (RACK1) was reported to competitively bind HIF-1α against heat shock protein 90 (HSP90) to induce oxygen-independent degradation of HIF-1α [46]. In addition, Garth Powis et al. [47] reported that hypoxia-associated factor(HAF) mediates HIF-1α degradation independent of pVHL or oxygen. Pharmacological depletion of Fe^2+^ also induces HIF because Fe^2+^ is a cofactor of PHD and FIH [35].

### 2.3. Functions of HIF

The major function of HIF depends on its transcriptional activity (Figure 1). HIF-α translocates into the nuclei and binds to HIF-β via the bHLH/PAS domains to form the HIF heterodimer. The heterodimerized HIF recruits coactivator p300 and CBP and specifically binds to the HRE [5′-RCGTG-3′ (R = A or G)] in the target gene promotor to regulate the transcription of a wide range of genes [13,35,40,48].

As a transcription factor, HIF regulates the expression of approximately 400 target genes in various biological processes [13,49]. Especially, HIF is a critical regulator in maintaining oxygen homeostasis and has a close relationship with the reactive oxygen species (ROS) signaling pathway, protecting organs and tissues against oxidative damages [50,51]. Under hypoxia, ROS can either promote HIF-1α transcription [52] or stabilize HIF-1α protein [53]. Conversely, HIF-1 activation may alleviate ROS production. Overexpression of HIF-1α was shown to protect against 1,4- benzoquinone-induced apoptosis by reducing ROS level in K562 cells [54]. Jiang et al. showed that HIF-1α stabilization by dihydrotanshinone I (DI) pretreatment has a cardio-protective effect after ischemic injury by inducing nuclear factor E2-related factor 2 (Nrf2) expression and consequent activation of antioxidant enzymes to reduce ROS [55].

In addition, HIF may affect autophagy through its regulation of BCL2 Interacting Protein 3 (BNIP3)-related pathway [56,57]. BNIP3 or BCL2 Interacting Protein 3 Like (BNIP3L) is transcriptionally upregulated by HIF during hypoxia. The induced BNIP3/BNIP3L then competes with B-cell lymphoma 2 (Bcl2) and B-cell lymphoma-extra large (BCL-xL) to bind with Beclin1. The dissociation of Beclin1 from Bcl2 or Bcl-xl may promote the activation of autophagy.

Under hypoxic conditions, HIF also promotes angiogenesis by up-regulating the genes encoding angiogenic growth factors and cytokines, which recruit and regulate bone marrow-derived angiogenic cells (BMDACs) [58]. Transgenic mice overexpressing HIF-1α have an increased level of vascular endothelial growth factor (VEGF), a key mediator of angiogenesis in normal and pathological conditions, and exhibit significant induction of hypervascularity [59]. In this regard, Rankin et al. [60] demonstrated that Hif-1α deficiency has little effect on VEGF expression, while HIF-2α activation is demonstrated to induce VEGF. Warnecke et al. [61] reported that both HIF-1α and HIF-2α may contribute to VEGF activation, although HIF-1α is significantly more effective.

EPO, a potent growth factor for erythropoiesis, is reported to be preferentially regulated by HIF-2α [61,62]. Under hypoxia conditions, HIF enhances EPO synthesis by promoting its transcription via direct binding to the HRE of EPO gene, which is coordinated with iron metabolism. HIF also regulates the bone marrow microenvironment to promote the maturation and proliferation of erythroid progenitor. Thus, HIF promotes erythropoiesis at multiple levels, and the therapeutic strategies of activating HIF are effective in the treatment of anemia [63].

HIF is also closely related to immune responses and inflammation induction. Under hypoxic stress, activated HIF plays an important role in regulating the survival and differentiation of immune cells and in controlling the expression of pro-inflammatory factors [28]. HIF-1α-dependent NF-κB activity is directly involved in the regulation of neutrophil survival under hypoxic stress [64]. Cramer et al. [65] reported that HIF-1α is crucial for energy metabolism and function of myeloid cell. Inactivation of HIF-1α markedly suppresses the motility, invasiveness, aggregation, and anti-bacterial efficiency of macrophages, while stabilization of HIF-1α by VHL inhibition causes significantly enhanced inflammation. Also, HIF-1α induction by hypoxia or lipopolysaccharide (LPS) stimuli promotes glycolysis in dendritic cell (DC), which results in DC maturation for innate immune response activation [66]. Meanwhile, LPS-induced HIF-1α can contribute to the development of sepsis by the induction of multiple inflammatory cytokines [67]. In addition, Kojima et al. [68] reported an essential role of HIF-1α in B cell development where HIF-1α deficiency leads to abnormal peritoneal B-1 cells, aberrant maturation of B-2 cells, and autoimmunity. HIF-1α was also reported to participate in myeloid-derived suppressor cells (MDSC)-mediated T cell activation via the transcriptional up-regulation of programmed death ligand 1 (PD-L1) [69].

In tumorigenesis, HIF-1 participates in glucose metabolism, cancer cell survival and proliferation, angiogenesis, as well as metastasis [70]. HIF-1 activation in tumor cells up-regulates the expression of some glycolytic enzymes and transporters, including 6-phosphofructo-2-kinase/fructose-2,6-biphosphatase 3 (PFKFB3), M2 isoform of pyruvate kinase (PKM2), and glucose transporter 1 and 3 (GLUT1, GLUT3) [71]. PFKFB3 accumulation has been found to protect against cisplatin-induced apoptosis via enhanced glycolysis when accumulated in cells [72]. Emerging evidence has identified GLUT1 and GLUT3 as two major contributors to the accelerated glucose metabolism in tumor cells. Up-regulated GLUT1 and GLUT3 expression is associated with tumor resistance during anti-cancer therapy [73]. In addition, Azoitei et al. [74] reported that PKM2 accumulates in hypoxic pancreatic tumors to promote HIF-1α-induced VEGF secretion via the NF-κB pathway, leading to enhanced angiogenesis. Overall, HIF plays a crucial role in tumor maintenance, progression, and treatment resistance. Thus, targeting HIF-1 has become a therapeutic strategy for tumor treatment [75].

In addition to the transcriptional regulation of gene expression, emerging evidence indicates that HIF can also function independently of its transcriptional activity. In various tumor cells, even without DNA binding domain, HIF-1α can promote the ubiquitination of Dicer to facilitate its degradation by autophagy-lysosomal pathway. The downregulation of Dicer by HIF leads to the decrease of multiple tumor suppressive microRNAs and enhanced tumor metastasis [76]. In acute myeloid leukemia, HIF-1α can stimulate cell differentiation without HIF-1β, implicating its non-transactivation function [77]. Hubbi et al. [78] have shown that HIF-1α can function as a inhibitor of DNA replication, which is independent of its transcriptional activity. Under hypoxic condition, HIF-1α directly binds to cell division cycle 6 (Cdc6) and promotes its interaction with the minichromosome maintenance complex, leading to suppressed firing of replication origins and cell cycle arrest. HIF-1α was also reported to induce cell cycle arrest via the activation of p21 by displacing cellular Myc (c-Myc) and preventing its inhibitory effect on p21 expression [79]. In addition, HIF-1α may translocate to mitochondria under oxidative stresses to suppress ROS generation, mitochondrial membrane potential loss, mitochondrial DNA-encoded gene expression, and cell death [80]. The mitochondrially translocated HIF-1α is associated with the outer membrane, indicating its non-transactivation function.

## 3. Cisplatin-Induced Nephrotoxicity

Cisplatin-induced nephrotoxicity, firstly reported in the clinical trials of cisplatin chemotherapy, is one of the major side effects that limit cisplatin’s use and therapeutic efficacy [5]. Patients with solid tumors and malignant lymphomas who received cisplatin treatment have shown significant kidney injury [81,82]. A retrospective cohort study has shown that in intravenous cisplatin-treated patients with head and neck cancer, esophageal cancer, or gastric cancer, 41 out of 182 patients develop cisplatin nephrotoxicity during the initial administration. 71 cases have been observed in the total 442 cycles, with 8 patients developing multiple episodes of nephrotoxicity [83]. Other studies have shown nephrotoxicity development during cisplatin treatment of different cancers including ovarian cancer [84,85], hepatocellular carcinoma [86], lung cancer [87], colon cancer [88], breast cancer [89], and others [90]. Though various clinical manifestations of cisplatin induced-nephrotoxicity have been observed, in this review we mainly focus on cisplatin-induced AKI and CKD.

### 3.1. Cisplatin-Induced AKI

Among the multiple renal manifestations induced by cisplatin, AKI occurs in 20–40% of cancer patients following cisplatin treatment [4]. In days after cisplatin exposure, these patients show decreased glomerular filtration rate (GFR), increased level of blood urea nitrogen (BUN) and serum creatine, as well as disruption of fluid and electrolyte homeostasis [91]. The mechanism of cisplatin-induced AKI is a very complex process, which is the focus of our discussion in the following sections.

#### 3.1.1. Cisplatin Uptake and Metabolism

The uptake and accumulation of cisplatin in proximal tubules, especially in the S3 segment, causes massive renal tubular damage [92]. Cisplatin enters the cells not only by passive diffusion through cellular membrane, but also by active transportation [91,93]. Two membrane transporters, the copper transport protein (Ctr1) and the organic cation transporter 2(OCT2), contribute to the uptake of cisplatin in tubular cells [94,95]. Once cisplatin enters kidney tubular cells, it goes through a series of bio-activation processes to generate various toxic metabolites. For example, γ-glutamyl transpeptidase (GGT) and cysteine-S-conjugate β-lyase may catalyze the reactions to generate glutathione-cisplatin conjugate, cysteinyl-glycine-conjugate, cysteine-conjugate, and reactive thiol. Blocking either GGT or cysteine-S-conjugate β-lyase results in reduced cisplatin-induced nephrotoxicity [96,97].

#### 3.1.2. Cisplatin Induced Cell Death

Cisplatin triggers the activation of multiple signaling pathways, which eventually results in cellular injury and death. The most well-known toxic effect of cisplatin is DNA damage [5,6]. After binding to DNA with high affinity, cisplatin causes cross-links between and inside the strands of DNA, and impedes DNA synthesis and replication, thereby leading to cell-cycle arrest and DNA damage response [98,99]. Cisplatin induces several types of renal tubular cell death, such as necroptosis, ferroptosis, and apoptosis.

Necroptosis, a programmed form of necrosis, is one of the major contributors of proximal tubular cell death in cisplatin-induced AKI. Cisplatin stimulates receptor-interacting protein 1 (RIP1)/receptor-interacting protein 1 (RIP3)/mixed lineage kinase domain-like protein (MLKL)-dependent necroptosis of tubular cells and induces pro-inflammatory cytokines release, and the latter is capable to further promote necroptosis [100]. Pharmacological [100,101,102] or genetic [100,103] inhibition of the RIP1/RIP3/MLKL pathway can ameliorate tubular cell damage, inflammatory responses, and cisplatin-induced AKI. Other strategies that alleviate necroptosis have also shown a protective effect on cisplatin induced AKI [104,105].

Ferroptosis is an iron-dependent form of nonapoptotic cell death characterized by the iron-catalyzed accumulation of lethal lipid ROS and differs from apoptosis, necrosis, and autophagy in morphological, biochemical, and genetic features [106]. Ferroptosis also plays a role in the pathogenesis of cisplatin-induced AKI and the inhibition of ferroptosis reduces cisplatin-induced AKI [107]. Lu et al. [108] have shown that Ras homolog enriched in brain (Rheb1) deletion in renal tubules promotes cisplatin-induced AKI partially through enhanced ferroptosis. Ferroptosis, featured by lipid peroxidation, can be attenuated by vitamin D receptor (VDR) activation, which also ameliorates cisplatin-induced AKI [109]. Similarly, other anti-ferroptosis agents that prevent lipid peroxidation and alleviate ferroptosis improve renal function, diminish renal tubular damage, and attenuate cisplatin-induced AKI [110].

Additionally, cisplatin has been well-known to activate various pathways of apoptosis to cause renal damage. Cisplatin evokes the extrinsic apoptotic pathway of death receptor by mediators including Fas and tumor necrosis factor α (TNF-α) [91,111,112]. Cisplatin also induces mitochondrial dysfunction to cause intrinsic apoptosis through caspase-9 activation [5,113,114]. In addition, cisplatin also promotes apoptosis by elevated caspase-12 activation via the endoplasmic reticulum (ER) stress pathway [115]. Apoptosis is a critical target for treatment of cisplatin-induced AKI due to its essential role in tubular cell death. Studies have reported that attenuated apoptosis is beneficial to cisplatin-induced AKI [90]. Kong et al. [116] has reported that EPO protects against cisplatin-induced AKI in rats by attenuating tubular cell apoptosis. Yang et al. [117] have reported that inhibition of apoptosis partially contributes to the reno-protective effect of Asiatic acid. However, although the pan caspase inhibitor zVAD-fmk has shown a reno-protective role against ischemic AKI mice by inhibiting apoptosis [118], it increases cisplatin-induced necrotic cell death and impairs autophagic flux, leading to aggravated kidney dysfunction in cisplatin-induced AKI [119].

#### 3.1.3. Other Mechanisms

The tumor suppressor p53 plays an essential role in cisplatin-induced cell death, due to its central role in DNA damage response, apoptosis induction, cell cycle arrest, and oncogene inactivation [91,99,120,121]. Autophagy is a cytoprotective mechanism in cisplatin-induced AKI, and the inhibition of autophagy worsens AKI [122,123]. Inflammation also contributes significantly to cisplatin-induced nephrotoxicity. By activating Toll like receptors (TLRs) via the secretion of damage-associated molecular pattern molecules (DAMPs), cisplatin induces the release of a broad range of chemokines and cytokines, which leads to the infiltration of inflammatory cells and increased inflammatory responses in kidney tissues [5,90,91,124]. Oxidative stress has been considered to be another important injurious factor in cisplatin-induced nephrotoxicity. Through the depletion of antioxidants (e.g., glutathione), defective mitochondrial respiratory chain, and cytochrome P450 effects in microsomes, cisplatin induces cellular ROS generation, which may further trigger apoptosis and promote inflammatory responses [5,91,125]. In addition, endothelial dysfunction and vascular injury may be part of the side effects of cisplatin, leading to insufficient renal blood flow and hypoxic condition in kidney [126,127]. Accumulating evidence has demonstrated that the kidney tubules are vulnerable to hypoxic injury, due to their low oxygen tensions resulted from the unique renal vascular architecture, low capacity of anaerobic energy generation, as well as high consumption of oxygen required for cellular activities [128]. Hypoxia in kidney after cisplatin injury may further augment tubular injury. Moreover, epigenetic alterations including DNA methylation, histone modification, and noncoding RNAs are involved in acute kidney dysfunction after cisplatin injury [90,129].

### 3.2. Cisplatin Induced CKD

AKI patients are commonly observed to suffer the loss of kidney function, which may be transient [92]. However, even after initial recovery, AKI is an important risk factor for the development of CKD [130]. Although a single high dose of cisplatin mainly induces AKI, it may also have long term effects on kidney and act as a high-risk factor for CKD development [7]. Emerging evidence indicates that over 50% of patients receiving cisplatin chemotherapy may suffer a CKD progression [131]. In addition, in clinical practice, cancer patients often receive relatively low doses of cisplatin for several cycles. Recent studies have begun to investigate the chronic effect of repeated low-dose cisplatin treatment on kidney [9,132,133,134,135,136,137,138]. Sharp et al. [133] have reported that when compared to the AKI model, repeated low dose of cisplatin significantly enhances inflammatory cytokines and chemokines but has a lower level of cell death. Meanwhile, elevated expression of fibrotic markers and interstitial fibrosis occur. Long-term observation for 6 months reveals that the mice in the repeated dosing model have glomerular pathologies and persistent endothelial dysfunction [137]. The development of CKD may have some variations between different animal strains [138]. Similarly, Torres et al. [134] have reported that mice receiving two doses of cisplatin with a 2-week interval between injections followed by long-term observation show significant body weight decline and loss of renal function, as well as other CKD features including endothelial rarefaction, myofibroblast proliferation, and macrophage infiltration. Notably, “atubular” glomeruli have been observed and considered to be associated with degenerating tubules. Similar results have been reported in a few other in vivo and in vitro studies [9,135]. Although the severity of impairment in these studies may vary due to the different doses of cisplatin, these evidences indicate that repeated low-dose cisplatin treatment indeed leads to CKD progression, which is characterized by declined renal function, reduced kidney weight, chronic interstitial inflammation and fibrosis, endothelial dysfunction, as well as morphological changes such as atubular glomeruli.

According to the recent studies, CKD development is mainly due to the maladaptive repair after kidney injury. Kidney has the capacity of repairing damaged tubules. Depending on the severity of initial injury, there are two categories of kidney repair, normal complete repair and maladaptive incomplete repair [139]. When the injury is mild to moderate, kidney may undergo complete or adaptive repair and the injured tubular epithelia can proliferate and regenerate to restore the normal structure and function of kidney [139,140]. However, when the injury is severe or prolonged, the kidney may go through incomplete or maladaptive repair. Under this circumstance, CKD progression is associated with impaired tubular regeneration, endothelial dysfunction and prolonged hypoxia, persistent inflammation, interstitial fibrosis, epigenetic alteration, activated renin-angiotensin-aldosterone-system (RAAS), mitochondrial dysfunction, and cellular senescence [141,142,143,144]. Some of these changes have been reported in cisplatin-induced CKD [9,135,145,146,147,148].

## 4. HIF in Cisplatin-Induced AKI

Over the past few decades, several studies demonstrated the evidence for a reno-protective role of HIF in cisplatin-induced AKI [17]. These studies are summarized in Table 1.

HIF induction in cisplatin-induced AKI has been reported by several studies though whether cisplatin can directly activate HIF is still controversial. Xu et al. [152] have reported the increases in renal HIF-1α mRNA in rats after cisplatin injection in a dose- and time-dependent manner. Meanwhile, multiple well-known HIF-targeted genes such as VEGF and Heme oxygenase-1 (HO-1) are also induced, indicating activation of the HIF pathway. Using “HIF-sensing” transgenic mice to reveal HIF activation, Tanaka et al. [16] have found increased activation of HIF-1 in renal tubules (mainly the proximal tubular cells in the outer medulla) and HIF-2 activation in peritubular area in rats after cisplatin injection. However, no HIF activation is observed in cultured proximal cell line of rat after cisplatin treatment [16]. Weidemann et al. failed to detect HIF activation either in vivo or in vitro [17]. Instead, cisplatin treatment may impair HIF activation as the downstream GLUT1 mRNA transcription is reduced in HKC-8 cells treated with cisplatin [17]. Thus, it is possible that in in vitro conditions, the existence of normal oxygen (21% O_2_ level usually used for cell cultivation) may prevent HIF activation with cisplatin injury. However, in in vivo conditions, cisplatin-induced vascular damage and renal blood flow reduction may break the balance between low delivery and high consumption of oxygen in renal tubules, leading to severe local hypoxia [127]. Though with some variations in different in vivo models, HIF is likely activated due to this local hypoxia after cisplatin injury.

Consistently, the protective effect of HIF activation has been reported in several studies. Tanaka et al. have shown that cobalt (HIF-1α inducer) attenuates cisplatin-induced AKI in rats. The protective role of HIF-1 has been further confirmed in vitro by HIF-1α siRNA or dominant negative HIF-1α expression [16]. Although HIF activation was not detected after cisplatin treatment by Weidemann et al., hypoxic preconditioning to induce HIF activation protected both cultured renal cells and kidneys in their study [17]. Their in vitro experiments indicated that the protection by hypoxic preconditioning is HIF-dependent as HIF-1α knockout abolishes the protective effect [17]. The reno-protection by HIF activation in cisplatin AKI models has been further confirmed in other studies using PHD inhibitor FG-4592, iron chelator deferiprone, HIf-1α transfected human adipose-derived stem cells, or PHD1 knockout [18,19,149,150,151]. Interestingly, cisplatin induced less apoptosis in hypoxic kidney cells than in normoxic cells, but this protective effect of hypoxia may not be directly related to HIF [17,153]. Our study indicates that this effect of hypoxia is related to P53 activation [153]. Overall, the in vivo and in vitro evidence implicates that HIF up-regulation can be an effective therapeutic strategy for preventing cisplatin-induced AKI.

**Figure 2:**
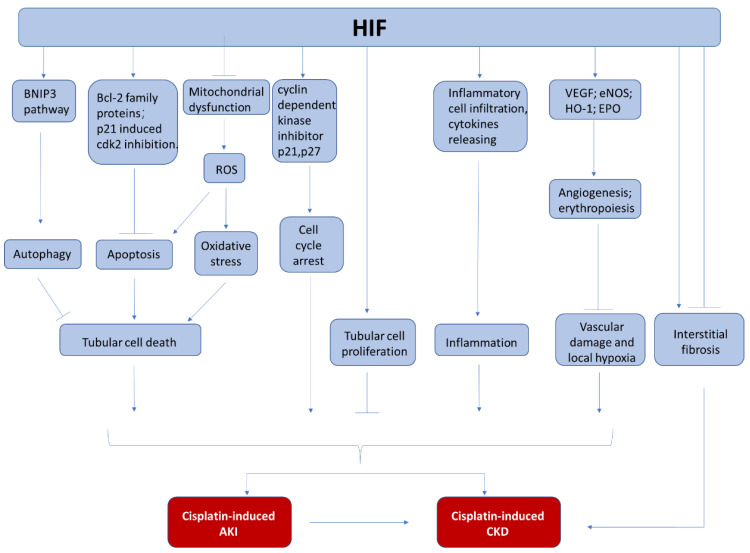
The involvement of HIF in cisplatin-induced nephrotoxicity. During cisplatin chemotherapy, HIF may protect kidneys from AKI and CKD by the inhibition of tubular cell death, the regulation of cell proliferation, the suppression of kidney inflammation, and the attenuation of vascular damage.

Currently, the underlying mechanism for the reno-protection of HIF in cisplatin induced nephrotoxicity has been explored but not completely understood (Figure 2). In most of the studies, the reduction of renal tubular apoptosis has been reported. The anti-apoptotic effect of HIF-1 activation is accompanied with increased expression of BCL-2 family proteins (MCl1, survivin) [19]. In addition, HIF activation is also associated with anti-oxidative effects, showing less lipid peroxidation [149], which may be related to ferroptosis inhibition. Furthermore, HIF activation may protect endothelial cells because angiogenesis-related genes such as VEGF may be induced [150,151]. Finally, the anti-inflammation effect of HIF activation may also contribute to its reno-protection. HIF upregulation by PHD inhibitor or HIF-1α overexpression can significantly reduce multiple cytokine release after cisplatin injury, which can lead to less inflammatory cell infiltration and reduced renal injury [18,151].

Finally, HIF also interacts with other pathways that are involved in the development and progress of cisplatin-induced AKI. Goda et al. [154] have found that hypoxia upregulates p27 and p21 in a HIF-dependent manner and the enhanced p27 expression ultimately prevents retinoblastoma protein (Rb) hyperphosphorylation, making HIF a crucial determinant of hypoxia-induced cell cycle arrest. Thus, HIF may participate in regulating cell cycle arrest in cisplatin-induced nephrotoxicity. Besides, p21 is proved to prevent cell apoptosis in cisplatin-induced AKI by directly inhibiting cyclin-dependent kinase 2 (cdk2), which may be another pathway for HIF to exert its protection [155,156]. In addition, HIF has been reported to activate autophagy through BNIP3 pathway in other systems [56,157], and autophagy has been shown to protect kidney from cisplatin injury [158,159,160,161,162], although it is unclear about the HIF/BNIP3 pathway regulation in cisplatin-induced AKI.

In conclusion, current evidence indicates that HIF activation is beneficial to the kidney during cisplatin chemotherapy. Further investigation on HIF-1 and its interaction with other cellular process may reveal deeper understanding and novel therapeutic targets of cisplatin-induced AKI.

## 5. HIF in Cisplatin-Induced AKI to CKD Progression

Numerous evidences have demonstrated that tubulointerstitial hypoxia is not only an essential contributor to AKI but also a key player in CKD. Chronic hypoxia is considered as a common pathological condition in CKD [10,163]. Though studies have revealed that multiple pathophysiological mechanisms are related to cisplatin-induced AKI to CKD progression [9,135,145,146,147,148], the investigation focusing on the role of HIF in this field is lacking. Nevertheless, HIF is a potential player in this process because of its essential role in hypoxia and its interactions with multiple pathophysiological procedures involved in the progression of CKD (Figure 2).

It is well-known that HIF-1α plays a crucial role in kidney repair in various types of CKD [10]. After kidney injury, the surviving epithelial tubular cells, which are commonly considered to be responsible for tubular regeneration, go through dedifferentiation, proliferation, and re-differentiation to replace the lost neighboring cells [164]. The disruption of balance between tubular cell death and proliferation may result in maladaptive repair and lead to CKD progression [10]. In cisplatin-induced AKI, HIF-1α affects tubular cell apoptosis via the regulation of Bcl-2 family members [19] and mitochondrial function [16]. Meanwhile, HIF also regulates tubular cell proliferation through different downstream pathways, either positively or negatively [10]. Although HIF activation is mainly to alleviate epithelial tubular cell death and promote cell proliferation and renal recovery after cisplatin-induced AKI, its effect on tubular cell repair in cisplatin-induced AKI to CKD progression remains unclear and warrants further investigation.

Oxidative stress has been shown to be a major injurious factor during CKD development. DNA damage by oxidative stress and the associated DNA damage response may prevent the cell cycle progression during repair and induce G2/M arrest [147] and senescence [148]. Chemical treatment by semicarbazide sensitive amine oxidase inhibitor (PXS-4728A) [135], superoxide dismutase (SOD) mimetic (GC4419) [146], or general antioxidants like N-acetylcysteine (NAC) [148] to suppress oxidative stress significantly reduces cisplatin-induced CKD progression. Considering the antioxidative function of HIF in cisplatin-induced AKI [149], it is possible that HIF may benefit the tubular repair and regeneration to alleviate CKD progression.

Yamaguchi et al. [34] discovered CEBPD as a novel regulator of HIF-1 in acute model of cisplatin-induced nephrotoxicity and in models of other acute and chronic hypoxic kidney injury. CEBPD is not only a transcription factor that transactivates HIF, but is also induced by inflammatory signals like interleukin 1β (IL–1β) via the NF-κB pathway. Thus, CEBPD can act as a mediator by which inflammation affects HIF-1 in both acute and chronic kidney diseases [34]. Conversely, HIF has a role in inflammatory infiltration and responses [28,65,165]. Inflammation is a well-known feature of maladaptive repair in the process of CKD [10]. These evidences suggest a cross talk between inflammation and HIF pathways. Emerging evidence indicates that repeated low-dose cisplatin treatment induces significant interstitial inflammation, which contributes to the progression of CKD [133,134,135,137]. Apparently, more work on HIF is needed to explore its exact role in chronic inflammation in cisplatin-induced AKI to CKD progression.

Renal interstitial fibrosis is a common final pathological feature of CKD. Studies have reported that repeated low-dose cisplatin administration leads to significant interstitial fibrosis, a sign of AKI to CKD progression [9,133,134,135]. Numerous studies have demonstrated that HIF, particularly HIF-1α, is a key regulator of renal interstitial fibrosis in the progression of CKD, though controversial opinions may exist on whether it promotes or suppresses renal fibrosis [166]. On one hand, HIF activation may attenuate fibrosis in various types of CKD models. Kapitsinou et al. [167] have showed pharmacological activation of HIF before ischemia/reperfusion injury (IRI) improves kidney fibrosis, while no beneficial effect is observed by post-injury activation. They have also reported that PHD inhibitors suppress interstitial inflammation and fibrosis by activating endothelial HIF-2 and promote renal recovery after kidney IRI [168]. Kobayashi H et al. [169] have shown that systemic HIF activation suppresses unilateral ureteral obstruction (UUO)-induced kidney inflammation and fibrosis in mice. Several other studies have also reported that pharmacological activation of HIF-1α alleviates interstitial fibrosis and improves kidney recovery in the rat remnant kidney (RK) model of CKD [170,171]. On the other hand, there are emerging evidences showing a profibrotic role of HIF activation. Kimura et al. [171] have found that stable tubular expression of HIF-1α promotes fibrosis in mouse RK model, while HIF1-α inhibition ameliorates UUO-associated renal fibrosis in mice. Liu et al. [172] have reported that HIF-1α transcriptionally upregulates p53 to promote G2/M cell cycle arrest and renal fibrosis in hypoxic tubular cells and in UUO mice. Consistently, genetic inhibition of HIF-1α shows attenuated fibrosis in angiotensin II-induced renal injury [173] and in chronic ischemic renal injury [174].The reason for the controversial effects of HIF activation on fibrosis remains unknown, but it may be related to the differential roles of HIF in different renal cell types or in different pathological stages (injury vs. repair). Further research focusing on HIF’s role in development of fibrosis in cisplatin-induced AKI to CKD progression may bring us deeper insight into the pathological mechanisms and potential intervening strategies.

Furthermore, HIF participates in the pathogenesis of CKD-related complications, such as anemia [175,176] and vascular calcification [177], which can affect the clinical outcome of CKD regardless of the initial causes of kidney damage. Recently, several clinical trials have confirmed the beneficial effects of PHD inhibitors in treating CKD-related anemia [175,176,177,178,179]. In CKD patients, the stabilization of HIF by PHD inhibitors can improve the EPO level and attenuate anemia, an observation leading to clinical use of PHD inhibitors. Recent studies also showed a new series of orally active PHD2 inhibitor, such as 15i [180] and 17 [179] can significantly improve cisplatin-induced anemia in mice without apparent toxicity. However, HIF induction has been reported to enhance vascular calcification, which may deteriorate CKD [177]

## 6. Therapeutic Potential of HIF in Cisplatin Chemotherapy

In view of the role of HIF in cisplatin-induced nephrotoxicity, targeting or activating HIF and its related pathways may be a therapeutic strategy. PHD-pVHL pathway plays a vital role in HIF regulation, and pharmacological or genetic inhibition of PHD activity is under intensive research in kidney diseases [18,45,167,168,181,182,183,184,185]. The role of regulating HIF in cisplatin-induced nephrotoxicity is summarized in Table 1; besides the evidence that HIF accumulation by PHD inhibitors treatment protects against cisplatin-induced nephrotoxicity [18], studies have reported beneficial effects of PHD inhibitors in other kidney diseases including ischemic AKI [167,168], diabetic nephropathy [182,183], obesity related kidney disease [181], chronic tubulointerstitial nephritis [184], and remnant kidneys models of CKD [186]. A great breakthrough in this field is that PHD inhibitors have been proved to have a therapeutic effect in anemia, a complication of CKD that contributes to poor clinical outcome [187]. These inhibitors block enzymatic activity of PHD to stabilize HIFs, whose accumulation leads to increased transcription and expression of EPO, followed by enhanced erythropoiesis to alleviate anemia in CKD patients [45,176]. Recently, Wu et al. [180] found that an orally active PHD2 inhibitor 15i can bring the hemoglobin in cisplatin-induced anemia mice to a normal level with no obvious toxicity observed. Zhang et al. [179] also discovered that another PHD2 inhibitor 17 can significantly improve cisplatin-induced anemia in mice with an excellent safety property. Compared with the conventional erythropoiesis-stimulating agents (ESAs), PHD inhibitors have less side effects and are more convenient to be administered orally [45]. To date, following successful clinical trials, several small-molecule PHD inhibitors, including roxadustat (FG-4592), daprodustat (GSK1278863), and vadadustat (AKB-6548), are being used clinically for the treatment of anemia in CKD patients [188]. Meanwhile, PHD inhibitors are reported to benefit cardiovascular disease in CKD patients through HIF, and HIF activation improves myocardial remodeling, atherosclerosis and vascular injury [189,190].

However, most of the current investigations focusing on HIF’s role in cisplatin induced nephrotoxicity were conducted in tumor-free models that only have cisplatin exposure to study HIF’s effect on the pathophysiological mechanisms, while in clinical settings, cisplatin chemotherapy is always given to patients with tumor burden. When considering therapeutic strategies of cisplatin-induced nephrotoxicity, attention should be paid to their effects on both reno-protection and tumor sensitivity to cisplatin. Amifosten, the FDA-approved agent for the prevention of cisplatin-induced AKI in patients with non-small cell lung cancer (NSCLC) and advanced ovarian cancer, reduces renal toxicities of repeated cisplatin administration, with no reduction in antitumor efficacy [191]. Consistent with the experimental evidence that magnesium supplement significantly improved cisplatin-induced AKI and reduced tumor growth in mice with tumor xenograft [84,88], clinical data showed it may have a role as a protectant in cisplatin-induced AKI [192]. Our previous work found that pharmacological or genetic inhibition of PKCδ enhanced the chemotherapeutic effects of cisplatin in several xenograft and syngeneic mouse tumor models, while protecting kidneys from nephrotoxicity [85]. Sánchez-González et al. [193] reported that in rat breast adenocarcinoma model, quercetin ameliorated cisplatin-induced nephrotoxicity without interfering with its tumor toxicity. In view of the above, HIF’s role in tumor sensitivity to cisplatin should also be considered seriously when HIF-regulating strategies are studied as combination treatment of cisplatin chemotherapy. Notably, although HIF activation may have protective effect on kidney during cisplatin chemotherapy, it may attenuate the anti-cancer effects of cisplatin in tumors [194]. Intratumoral hypoxia is a common feature of solid tumors where vascular oxygen delivery cannot meet the oxygen consumption of rapidly growing cancer cells [194]. Generally, intratumoral hypoxia, together with functional alterations of tumor suppressor genes (most importantly, VHL) and oncogenes, upregulates HIF activity, which regulate multiple pathological processes and eventually result in the resistance to chemotherapy, radiation therapy, and targeted therapy [195,196]. Studies have revealed HIF overexpression in multiple tumors [20,197] and found that HIF is closely related to tumor resistance to cisplatin [198,199,200,201,202]. Zhang et al. [200] reported that HIF-1α may act as a mediator of the interaction between p53 and RAS signaling to participate in the cisplatin resistance in ovarian cancer and that silencing HIF-1α significantly decreased the cellular sensitivity to cisplatin. Keremu et al. [201] found dramatically elevated HIF-1α expression in cisplatin-resistant osteosarcoma cells under hypoxia. The inhibition of HIF-1α by miR-199a overexpression can sensitize the response of cisplatin-resistant cells. Ai et al. [202] reported that overexpression of degradation-resistant HIF-1α suppresses cisplatin-induced apoptosis while genetic knockdown of HIF-1α or pharmacological promotion of HIF-1α degradation enhances the response to cisplatin in ovarian cancer cells. Kizaka-Kondoh et al. [203] have demonstrated that HIF-1 activity is related to the invasion and metastasis of pancreatic cancer and the selective killing of HIF-1-actived cells via apoptosis by POP33 leads to suppressed peritoneal dissemination of cancer cells and improved mouse survival. Liu et al. [204] found that Oroxylin A directly binds to the bHLH-PAS domain of HIF-1α to prevent hypoxia-induced xeroderma pigmentosum group C (XPC) transcription, attenuating the cisplatin resistance in NSCLC. Parmakhtiar et al. [205] revealed that topotecan-induced HIF inhibition restores cisplatin and paclitaxel sensitivity in ovarian cancer via enhanced p53-mediated apoptosis. More studies showing HIF’s involvment in cancers during cisplatin chemotherapy are summarized in Table 2. In general, inhibiting HIF has been shown as a powerful strategy to reinforce anti-cancer efficiency of chemotherapeutic strategies including cisplatin [194,195,196,206]. Thus, in cisplatin chemotherapy, activating HIF is a double-edged sword. The failure to balance its effects on cisplatin’s anti-tumor function and nephrotoxicity may limit its application in cisplatin-induced AKI or CKD. For example, Erez et al. [207] reported that the overexpression of mouse PHD1 in colon carcinoma cells suppresses tumor growth by destabilizing HIF-1α. Bordoli et al. [208] demonstrated that PHD2 inhibition promotes tumor growth and that human biopsies with low-PHD2 protein expression level correlates significantly with shorter surviving time of breast cancer patients. Therefore, the clinical application of PHD inhibitors to reduce the side-effect of cisplatin requires further investigation and evaluation. A possible strategy is to develop drug delivery methods that can specifically regulate HIF activity in the kidney without systemic influence. Potential strategies for restrictive renal delivery have been studied, such as protein-based and peptide-based carriers, water-soluble polymeric carriers, small-molecule prodrugs, and nanoparticles [209]. However, renal specific HIF-regulating drug delivery has not been reported in the treatment of cisplatin-induced nephrotoxicity yet.

Meanwhile, because PHD has 3 isoforms (PHD1, PHD2, PHD3) that have different expression patterns and HIF-α targeting specificity in different cell types [10], cell type restricted PHD isoform-specific inhibitory strategy should also be taken into consideration for HIF regulation in cisplatin chemotherapy. Several studies have reported that endothelial PHD2 inhibition induced HIF-2α activation. It not only inhibits the intravasation and metastasis of cancer cells by normalizing the endothelial lining of tumor, improving the tumor perfusion, and increasing the chemotherapeutic drug delivery without affecting drug level in normal organs, but also alleviates the oxidative response to protect against cisplatin-induced nephrotoxicity and doxorubicin-induced cardiotoxicity [149,219]. However, further research is still needed to verify and extend these observations, especially examination of the long-term effect on cisplatin-induced CKD progression.

In addition, HIF-targeted therapies may enhance the efficacy of immunotherapies. HIF inhibition by topotecan can enhance the anti-tumor activity of bevacizumab in U251-HRE glioblastoma xenografts [220]. Kheshtchin et al. [221] reported that the combination of DC-based vaccination and PX-478, an inhibitor of oxygen-sensitive HIF-1α, enhances T cell effector functions and leads to inhibited tumor growth and enhanced survival in breast cancer mouse model. However, a recent clinical trial for NSCLC using the combination of evofosfamide, a hypoxia-targeted pro-drug, and tarloxotinib, has been stopped early due to futility [222]. Overall, it remains possible that the combination of HIF targeting therapy with immunotherapies may be a more effective anti-tumor strategy to replace the toxic therapies such as cisplatin in the future.

In conclusion, when considering therapeutic strategies activating or targeting HIF in cisplatin-induced nephrotoxicity, more comprehensive and rigorous work is still needed to identify novel chemicals or drug delivery techniques to achieve the maximal reno-protective effect without diminishing the anti-tumor efficacy of cisplatin.

## 7. Conclusions

In this review, we summarized the recent findings about HIF and cisplatin-induced acute and chronic nephrotoxicity and discussed the function of HIF in the injury development. The critical role of HIF in cisplatin nephropathy and the availability of potential clinical treatment such as PHD inhibitors make it a promising candidate for future therapeutics. However, the beneficial effect of HIF especially for cisplatin induced CKD is still waiting to be clarified by future study.

## Figures and Tables

**Table 1:** Summary of studies about the role of HIF in cisplatin-induced AKI.

Number	Model	Strategies for HIF Regulation	Involved HIF Isoforms	Is HIF Activated or Inhibited	Effects	Underlying Mechanisms	Reference
1	Rats	cobalt	HIF-1 and HIF-2,	activated	attenuate AKI	apoptosis reduction via regulating mitochondrial pathways	[16]
	IRPTC,		HIF-1	activated	improve IRPTC survival		
2	Rats;	Carbon monoxide preconditioning	HIF-1	activated	attenuated AKI	apoptosis reduction	[17]
	HCK-8 cells		HIF-1	activated	reduced apoptosis, increased proliferation		
3	Mice;	PHD inhibitor FG-4592	HIF-1	activated	attenuated AKI	apoptosis reduction, ameliorated inflammation	[18]
	HK-2 cells		HIF-1	activated	reduced apoptosis		
4	Rats	deferiprone	HIF-1	activated	attenuated AKI	reduce apoptosis with increased Mcl1 and survivin expression	[19]
5	Mice	EC-specific Phd2+/− mice	HIF-1, HIF-2	activated	attenuated AKI	induced antioxidative response	[149]
6	HK-2 cells	lentivirus-mediated HIF-1α-transfected hASCs	HIF-1	activated	reduced apoptosis and improved cellular morphology	reduced apoptosis	[150]
7	Mice	lentivirus-mediated HIF-1α-transfected hASCs	HIF-1	activated	attenuated AKI	reduced apoptosis, ameliorated inflammation	[151]

IRPTC, immortalized rat proximal tubular cells; HCK-8, human renal proximal tubular cell line; HK-2, human proximal tubule epithelial cells; Mcl1, Myeloid cell leukemia-1; EC, endothelial cell; hASCs, Human adipose-derived stem cells.

**Table 2:** Summary of studies about therapeutic potential of HIF in cisplatin chemotherapy.

Number	Model	Strategies for HIF Regulation	Involved HIF Isoforms	Is HIF Activated or Inhibited	Effects	Underlying Mechanisms	Reference
1	Ovarian cancer cell lines	HIF-1α SiRNA; 1-methyl-1, 9 PA	HIF-1α	inhibited	increased cancer cell sensitivity to cisplatin	regulation of aerobic glycolysis and mitochondrial oxidative phosphorylation; induced apoptosis through ROS overproduction	[202]
2	Ovarian cancer cell lines	HIF-1α SiRNA	HIF-1α	inhibited	increased cancer cell sensitivity to cisplatin	regulation of the interaction between p53 and RAS signaling and dysregulation of apoptosis and autophagy	[200]
	mice with ovarian cancer cell tumor xenograft			inhibited	inhibited tumor growth		
3	Ovarian cancer cell lines	Topotecan	HIF-1α	inhibited	reversed hypoxia-induced cisplatin and paclitaxel resistance	restored p53 transcriptional activity, downregulated ABCB1/ABCB5 cell surface expression	[205]
4	Lung adenocarci-noma Cells	HIF-2α SiRNA	HIF-2α	inhibited	increased cancer cell sensitivity to cisplatin	decreased the expression of P-glycoprotein 1	[210]
5	Human lung adenocarci-noma cell lines	HIF-1α SiRNA	HIF-1α	inhibited	increased cancer cell sensitivity to cisplatin	decreased MDR1 and MRP expression	[211]
6	Human lung adenocarci-noma cell lines	HIF-1α SiRNA	HIF-1α	inhibited	increased cancer cell sensitivity to cisplatin	unsure, possibly related to mechanisms other than MDR1 gene regulation	[212]
7	mice with tumor xenograft from human ESCC cell	HIF-1α SiRNA	HIF-1α	inhibited	inhibited tumor growth	induced cell apoptosis	[213]
8	Human NSCLC cell lines	Panobinost-at	HIF-1α	inhibited	inhibited cell proliferation and viability; suppressed growth of multicellular spheroids	more chromatin fragmentation and induction of apoptosis	[214]
9	Human prostate cancer cell	HIF-1α SiRNA	HIF-1α	inhibited	inhibited cell viability, proliferation, and colony formation capability	induced apoptosis through ROS overproduction	[215]
	mice with tumor xenograft from human prostate cancer cell				inhibited tumor growth		
10	Human NSCLC cell lines	Oroxylin A	HIF-1α	inhibited	increased cancer cell sensitivity to cisplatin	inhibited nucleotide excision repair through suppressing XPC transcription	[204]
	mice with tumor xenograft from human NSCLC cell				reduced tumor growth		
11	Human ovarian cancer line	Noscapine	HIF-1α	inhibited	increased cancer cell sensitivity to cisplatin	downregulated HIF-1 transcriptional activity and MDR1 overexpression	[216]
12	Human NPC cell lines	Evofosfami-de	HIF-1α	inhibited	exhibited hypoxia-selective cytotoxicity and increased cancer cell sensitivity to cisplatin	induced G2 cell cycle arrest and DNA damage	[217].
	Tumor-bearing mice with xenograft derived from human NPC cell lines				inhibited tumor growth		
13	Chondrosarcoma cell lines	EPAS1 shRNA	HIF-2α	inhibited	decreased sphere-forming potential, clonogenicity, and proliferative capacity; suppressed cell invasiveness	induced cell apoptosis	[218]
	Chondrosarcoma cell lines	HIF-2α inhibitor TC-S7009			blocked sphere formation, clonogenicity, invasive phenotypes, and matrix-degrading activity		
	Tumor-bearing mice with xenograft derived from chondrosarcoma cell lines				nearly abolished invasive outgrowth and profound suppression of metastasis		
14	Tumor-bearing mice with xenograft derived from melanoma cells	EC-specific Phd2+/− mice	HIF-1, HIF-2	activated	normalized tumor vessels; inhibited tumor growth, increased tumor sensitivity to cisplatin	increased vessel perfusion and drug delivery.	[149]

1-methyl-1, 9 PA, 1-methyl-1, 9-pyrazoloanthrone; ABCB1, ATP Binding Cassette Subfamily B Member 1; ABCB5, ATP Binding Cassette Subfamily B Member 5; MDR1, multidrug resistance-1; MRP, multidrug resistance-associated protein; ESCC, esophagus squamous cell carcinoma; NPC, nasopharyngeal carcinoma; EPAS1, Endothelial PAS Domain Protein 1.

## Data Availability

Data sharing not applicable.
